# Development and Validation of a Novel Mitochondrion and Ferroptosis-Related Long Non-Coding RNA Prognostic Signature in Hepatocellular Carcinoma

**DOI:** 10.3389/fcell.2022.844759

**Published:** 2022-08-12

**Authors:** Wuzheng Xia, Cong Zeng, Zehao Zheng, Chunwang Huang, Yu Zhou, Lan Bai

**Affiliations:** ^1^ Guangdong Provincial Key Laboratory of Gastroenterology, Department of Gastroenterology, Nanfang Hospital/The First School of Clinical Medicine, Southem Medical University, Guangzhou, China; ^2^ Department of Organ Transplant, Guangdong Provincial People’s Hospital, Guangdong Academy of Medical Sciences, Guangzhou, China; ^3^ Department of General Practice, Hospital of South China Normal University, Guangzhou, China; ^4^ Department of General Surger, Shantou University of Medical College, Shantou, China; ^5^ Department of Ultrasound, Guangdong Provincial People’s Hospital, Guangdong Academy of Medical Sciences, Guangzhou, China; ^6^ Department of Pancreatic Surgery, Department of General Surgery, Guangdong Provincial People’s Hospital, Guangdong Academy of Medical Sciences, Guangzhou, China

**Keywords:** lncRNAs, mitochondrion, ferroptosis, signature, hepatocellular carcinoma

## Abstract

Mitochondrion and ferroptosis are related to tumorigenesis and tumor progression of hepatocellular carcinoma (HCC). Therefore, this study focused on exploring the participation of lncRNAs in mitochondrial dysfunction and ferroptosis using public datasets from The Cancer Genome Atlas (TCGA) database. We identified the mitochondrion- and ferroptosis-related lncRNAs by Pearson’s analysis and lasso-Cox regression. Moreover, real-time quantitative reverse transcription PCR (RT-qPCR) was utilized to further confirm the abnormal expression of these lncRNAs. Based on eight lncRNAs, the MF-related lncRNA prognostic signature (LPS) with outstanding stratification ability and prognostic prediction capability was constructed. In addition, functional enrichment analysis and immune cell infiltration analysis were performed to explore the possible functions of lncRNAs and their impact on the tumor microenvironment. The pathways related to G2M checkpoint and MYC were activated, and the infiltration ratio of regulatory T cells and M0 and M2 macrophages was higher in the high-risk group. In conclusion, these lncRNAs may affect mitochondria functions, ferroptosis, and immune cell infiltration in HCC through specific pathways, which may provide valuable insight into the progression and therapies of HCC.

## Introduction

Hepatocellular carcinoma (HCC) accounts for 80% of the primary liver tumors and is one of the most common malignant cancers in the world (Petrick et al., 2016). From 1990 to 2017, the mortality of HCC in China increased by 50% (from 20 to 30 death per 100,0000) (Zhou et al., 2019). Surgical therapies (included surgical resection and liver transplantation) and tumor ablation are recommended as potential curative methods by several guidelines (Heimbach et al., 2018; Zhou et al., 2019; Benson, 2021). Most of the treatments can achieve excellent long-term survival but still remained unsatisfactory (Ishizawa et al., 2008; Bruix et al., 2016; Lencioni et al., 2016). Therefore, it is urgent to figure out the new therapeutic targets for hepatocellular carcinoma.

As a vital cellular organelle, the mitochondrion plays an important role in several crucial cellular activities, such as biosynthesis, signaling, and apoptosis (Zong et al., 2016; Yuan et al., 2020). Studies have shown that the mitochondrion can also have pivotal influence in cell fate decision (Shyh-Chang et al., 2013; Bahat and Gross, 2019). Moreover, current evidences indicate that mitochondrial defects are related to tumor growth and cell proliferation (Zheng et al., 2013; Zong et al., 2016; Li et al., 2020). Thus, mitochondrion and mitochondrial functions are regarded as potential therapeutic targets for several malignancies.

Dysfunction of iron metabolism is associated with tumorigenesis and tumor cell proliferation (Manz et al., 2016). Moreover, excessive intracellular iron accumulation and massive lipid peroxidation can impact the fate of the cell and lead to ferroptosis (Gao et al., 2019; Li et al., 2021) Ferroptosis is an iron-dependent and non-apoptotic-regulated cell death (Stockwell et al., 2017; Tang et al., 2021). At present, inducing ferroptosis of tumor cells is considered to be one of the promising directions for the treatment for several tumors (Hassannia et al., 2019).

Long noncoding RNAs (lncRNAs) are a heterogeneous group of non‐protein‐coding transcripts with lengths of greater than 200 nucleotides (Prensner and Chinnaiyan, 2011; Dhanoa et al., 2018). LncRNAs are broadly classified into sense intronic RNA, antisense RNAs, long intergenic RNAs (lincRNAs), and bidirectional RNAs (Ahadi, 2021). LncRNAs can promote tumorigenesis, cell proliferation, migration, metastasis, and other important biological processes by regulating gene expression on epigenetic modification (Nandwani et al., 2021). A recent study has found that lncRNA MT1DP elevates ferroptosis *via* downregulating NRF2 in NSCLC cells (Gai et al., 2020). Another study shows that lncRNA MALAT1 regulates metabolic reprogramming through mitochondrial metabolism (Zhao et al., 2021). These research studies indicate that lncRNAs may have diagnostic and prognostic potentials for HCC patients (Lanzafame et al., 2018; Zhang et al., 2020). However, it remains unclear about how the lncRNAs affect the HCC cell fate by regulating the mitochondrial defects and ferroptosis.

In this study, we focused on exploring the participation of lncRNAs in mitochondrial dysfunction and ferroptosis. We identified eight mitochondrion and ferroptosis-related lncRNAs by Pearson’s analysis, Lasso–Cox regression, and RT-qPCR. Based on these lncRNAs, the MF-related lncRNA prognostic signature (LPS) was constructed. This LPS had very outstanding stratification ability and prognostic prediction capability. In addition, function enrichment analysis and immune cell infiltration analysis were performed to explore the possible functions of lncRNAs and their impact on the tumor microenvironment.

## Materials and Methods

### Datasets and Patients

The hepatocellular carcinoma RNA transcriptome raw count data (LIHC) and their clinicopathological characteristic information were downloaded from The Cancer Genome Atlas database (TCGA, https://portal.gdc.cancer.gov/). Those patients were included who (1) only underwent surgical resection; (2) pathologically diagnosed HCC; (3) survival longer than 30 days were included in this study. The patients from TCGA were randomly divided at a ratio of 2:1 into the training set (*n* = 230) and validation set (*n* = 118). The clinicopathological characteristic information for the TCGA LIHC including in this study is listed in Table1.

**TABLE 1:** Clinical characteristics of HCC patients in the training cohort and validation cohort.

Variables	No. of patients	
	Training	Validation
Gender		
Male	158	78
Female	72	40
Age		
≥60	128	59
<60	102	59
Tumor stage		
Stage I	103	59
Stage II	49	29
Stage IIII	58	24
Stage IV	3	1
unknown	17	5
T stage		
T1	107	63
T2	56	30
T3	55	22
T4	10	3
N stage		1
0	158	85
1	1	2
unknown	71	31
M stage		
M0	162	86
M1	3	1
unknown	65	31

### Mitochondrion and Ferroptosis-Related Long Non-Coding RNA

The GENCODE website is an online database that integrates human and mouse gene annotations, including protein-coding RNAs, non-coding RNAs, and pseudogenes (Uszczynska-Ratajczak et al., 2018; Frankish et al., 2019). After downloading the lncRNA annotation file from the GENCODE website and recognizing the Ensemble ID of the gene in the TCGA LIHC dataset, all the lncRNAs were extracted. Differentially expressed lncRNAs were identified with the help of R packages *Deseq2* (Love et al., 2014). Cox proportional hazards regression was used to evaluate the prognostic value of these differentially expressed lncRNAs. According to previous articles, 1136 mitochondrial genes and 116 ferroptosis genes were selected (Supplementary Table S1), and their expression values were extracted from the TCGA LIHC dataset (Du and Zhang, 2020; Chen et al., 2021a; Rath et al., 2021; Zhou et al., 20212021). The R package *corrplot* was used to evaluate the correlations between genes and lncRNAs and calculated the correlation coefficient and *p* value. In this study, MF-related lncRNAs were defined as absolute correlation coefficient >0.5 and *p* value < 0.05.

### Constructing and Validating the Mitochondrion and Ferroptosis-Related Long Non-Coding RNAs Prognostic Signature

With the help of *Glmnet* and *survival* R packages (Engebretsen and Bohlin, 2019), eight MF-related prognostic lncRNAs were identified by least absolute shrinkage and selection operator (LASSO)-Cox regression analysis and RT-qPCR from 48 MF-related lncRNA and a MF-related LncRNA prognostic signature (LPS) was constructed (Tibshirani, 1997). The MF-related LPS risk score calculation formula was listed as follows:

$$Riskscore=\sum_{i=0}^{n}\beta i*xi$$

βi was the coefficient and the Xi was the expression of lncRNAi. Based on this formula, the risk score was calculated for each HCC patient.

Multiple Cox regression was utilized to identify the independent risk factors. The discrimination performance of the MF-related LPS was assessed by the area under the receiver operating characteristic (ROC) curve (AUC). Log-rank tests and Kaplan–Meier analyses were performed to assess the predictive ability and stratification ability of MF-related LPS and the independent risk factors. Overfit bias was decreased by bootstrap validation including 1000 resamples.

### Function Enrichment Analysis for the DEG Between the High-Risk and Low-Risk Group

To explore the mechanisms of different prognosis between low-risk and high-risk groups, differential analysis was performed based on the “Deseq2” package and only when the adjusted *p*-value <0.05 and | logarithmic fold-change (logFC)| >1.5 were statistically significant (Yang et al., 2019). Gene Ontology (GO), Kyoto Encyclopedia Genes and Genomes (KEGG), and gene set enrichment analysis of the DEGs were performed by R package cluster Profiler (Yu et al., 2012). We investigated the biological meaning of the common DEGs thorough GO analyses. It focused on the biological process and cellular components (CC). Kyoto Encyclopedia Genes and Genomes (KEGG) and gene set enrichment analysis (GSEA) helped us identify and evaluate the potential mechanisms and pathways.

### Correlation Analysis of the Mitochondrion and Ferroptosis-Related Prognostic Long Non-Coding RNA Signature and Immune Cell Infiltration

To assess the relationship between immune cell infiltration and our MF-related LPS, we performed the ESTIMATE analysis, EPIC analysis, and CIBERSORT algorithm (Chen et al., 2018; Racle and Gfeller, 2020). In addition, the Wilcoxon signed rank test was used to evaluate the proportion of immune cells between the high- and low-risk groups.

### Tissue Specimen and Real-Time Quantitative Polymerase Chain Reaction

Thirty pair samples of hepatocellular carcinoma and adjacent normal tissues were collected from Guangdong Provincial People’s Hospital (GDPH). The samples were immediately frozen after surgical resection and preserved in liquid nitrogen at −80°C. This study is approved by the Research Ethics Committee of Guangdong Provincial People’s Hospital.

Real-time quantitative polymerase chain reaction (RT-qPCR) was utilized to evaluate the lncRNA expression. All the samples were extracted using TRIzol reagent RNA TRIpure reagent (Aidlab Biotechnologies, RN01). cDNA was prepared using RevertAid Reverse Transcriptase (Thermos Fisher, United States). Real-time quantitative PCR was performed by the AceQ Universal SYBR qPCR Master Mix Kit. GAPDH was used for internal control. Each sample was tested in triplicate at least three times. All fold-changes were calculated through relative quantification 2^[(GAPDHCq) − (lncRNACq)]. The detailed primers sequences are provided in Supplementary Table S2.

### Statistical Analyses

Most statistical analyses were performed by R version 4.0.0 software (http://www.r-project.org/). The Deseq2 package was used to identify the differentially expressed lncRNA in the TCGA LIHC dataset. Cutoff values for the MF-related prognostic lncRNA and the nomogram were all defined by the receiver operating characteristic curve (ROC) analyses. Sangerbox, a useful and free online data analysis platform (http://www.sangerbox.com/tool), was used to analyze the correlation between the expression of 8 MF-related lncRNAs and clinicopathological factors. Kaplan–Meier analysis was used to compare the overall survival between the high-risk group and low-risk group. Univariate and multivariate Cox proportional hazards models were used to determine significant prognostic factors. The ROC curve was used to evaluate the predictive efficiency of the 8 MF-lncRNA. The figures of Kaplan–Meier analysis and ROC cures were drawn by Sangerbox platform. A *p* < 0.05 was considered statistically different.

## Results

### Patient Clinicopathologic Characteristics

**FIGURE 1:**
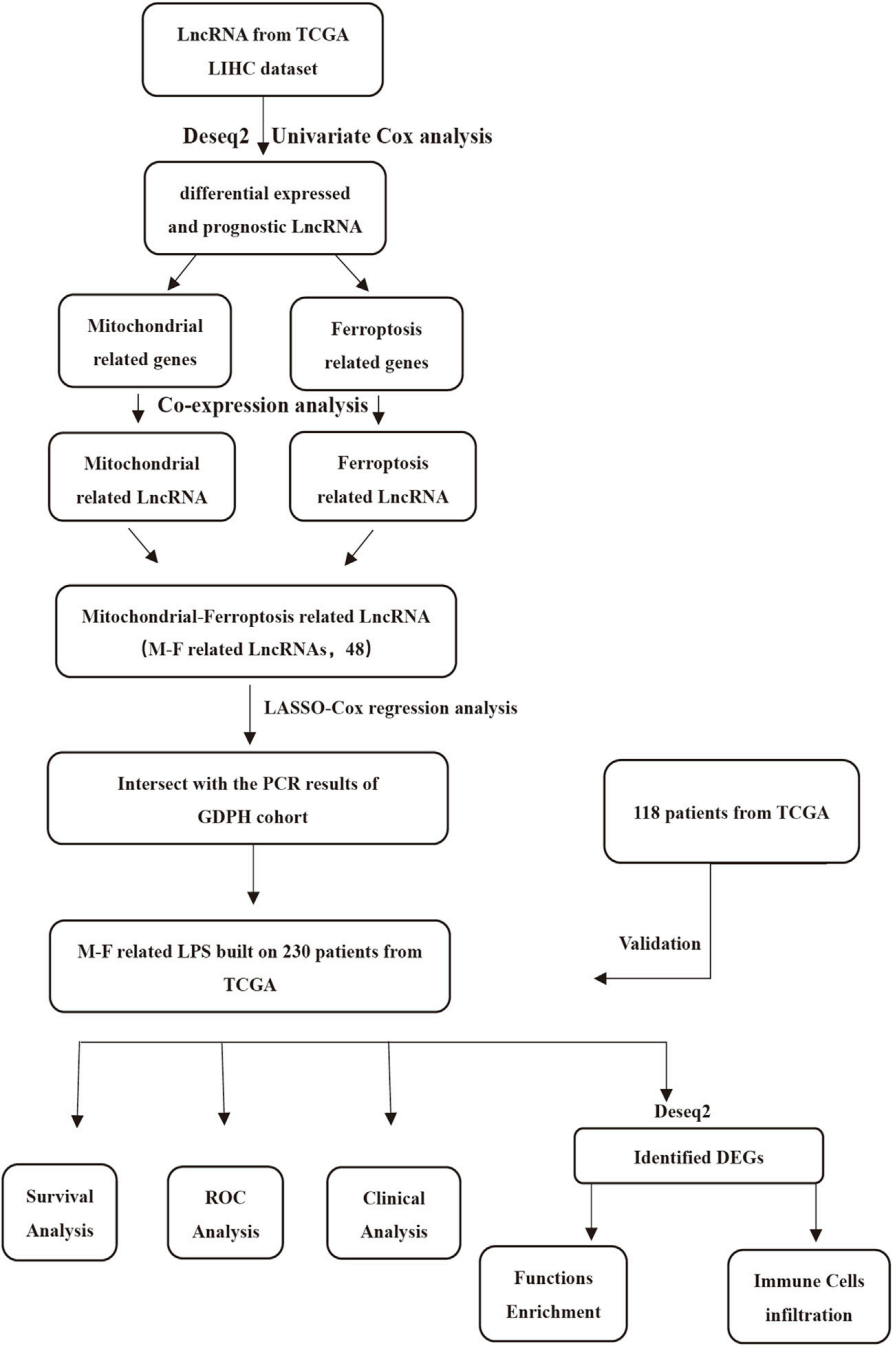
Study flow chart displays the work process of this project.

The flow chart (Figure 1) displays the work process of this project. Based on the criteria, 348 patients from the TCGA LIHC dataset were included in our study. The clinical data showed that the training set included 158 males and 72 females, while the validation set included 78 males and 40 females. The patients’ age of the training set and validation set ranged from 16 to 90. The overall survival of all the patients was longer than 30 days. Approximately, 66.1% patients in training cohorts and 74.6% patients in validation cohorts were at tumor stage I–II. Patient clinicopathologic characteristics are listed in Table 1.

### Identification of the Mitochondrion and Ferroptosis-Related in LIHC Patients

**FIGURE 2:**
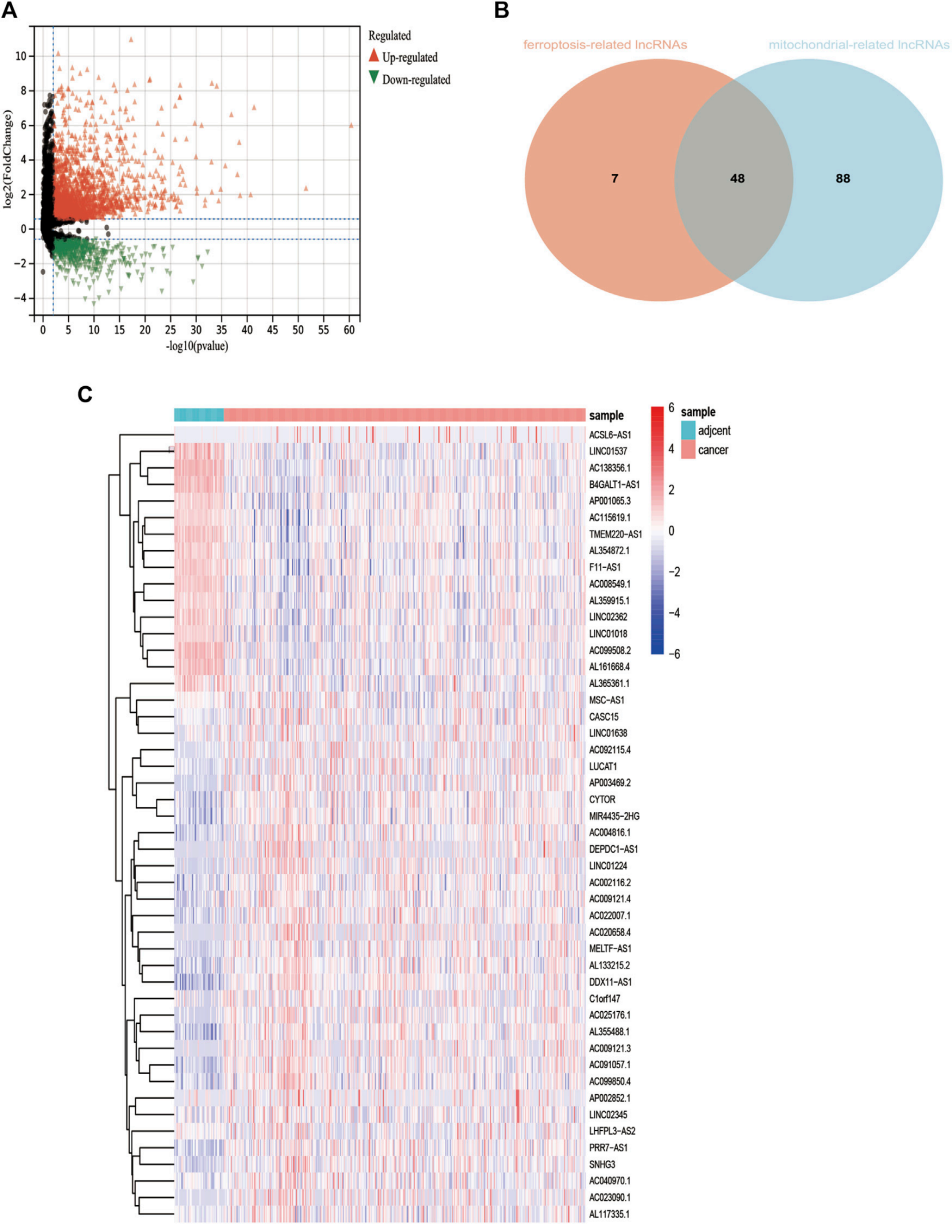
Identification of differentially expressed mitochondrial-related and ferroptosis-related lncRNA. **(A)**: Differential expressed lncRNAs based on TCGA. **(B)**: 48 mitochondrial- and ferroptosis-related lncRNAs. **(C)**: Heatmap of 48 mitochondrial- and ferroptosis-related lncRNAs *p < 0.05.**p < 0.01.***p < 0.001.***p < 0.0001.

The expression value of 12,079 LncRNAs was extracted from the TCGA LIHC dataset. We identified 2248 lncRNAs (1829 upregulated and 419 downregulated) that were differentially expressed in the TCGA-LIHC dataset (Figure 2A)**.** Univariate Cox regression analysis was performed to further estimate the impact of all the lncRNAs on the overall survival of LIHC patients in TCGA. Approximately, 404 differentially expressed and potential prognostic lncRNAs were screened out**.** By using R package corrplot, Pearson’s correlation analysis was performed to calculate the correlations between the expression of mitochondrial genes or ferroptosis genes and differentially expressed and prognostic LncRNA in the TCGA dataset. The lncRNA whose | Pearson R| *>* 0.5 and *p <* 0.05 was regarded as a mitochondrial-related or ferroptosis-related lncRNA. Subsequently, Venn analysis between the differential and prognostic mitochondrial-related lncRNAs and differential and prognostic ferroptosis-related lncRNAs was performed, generating the common lncRNAs (Figure 2B). Finally, we obtained 48 lncRNAs which were significantly correlated with mitochondrial-related and ferroptosis-related lncRNAs (MF-related lncRNAs, Figure 2C).

**FIGURE 3:**
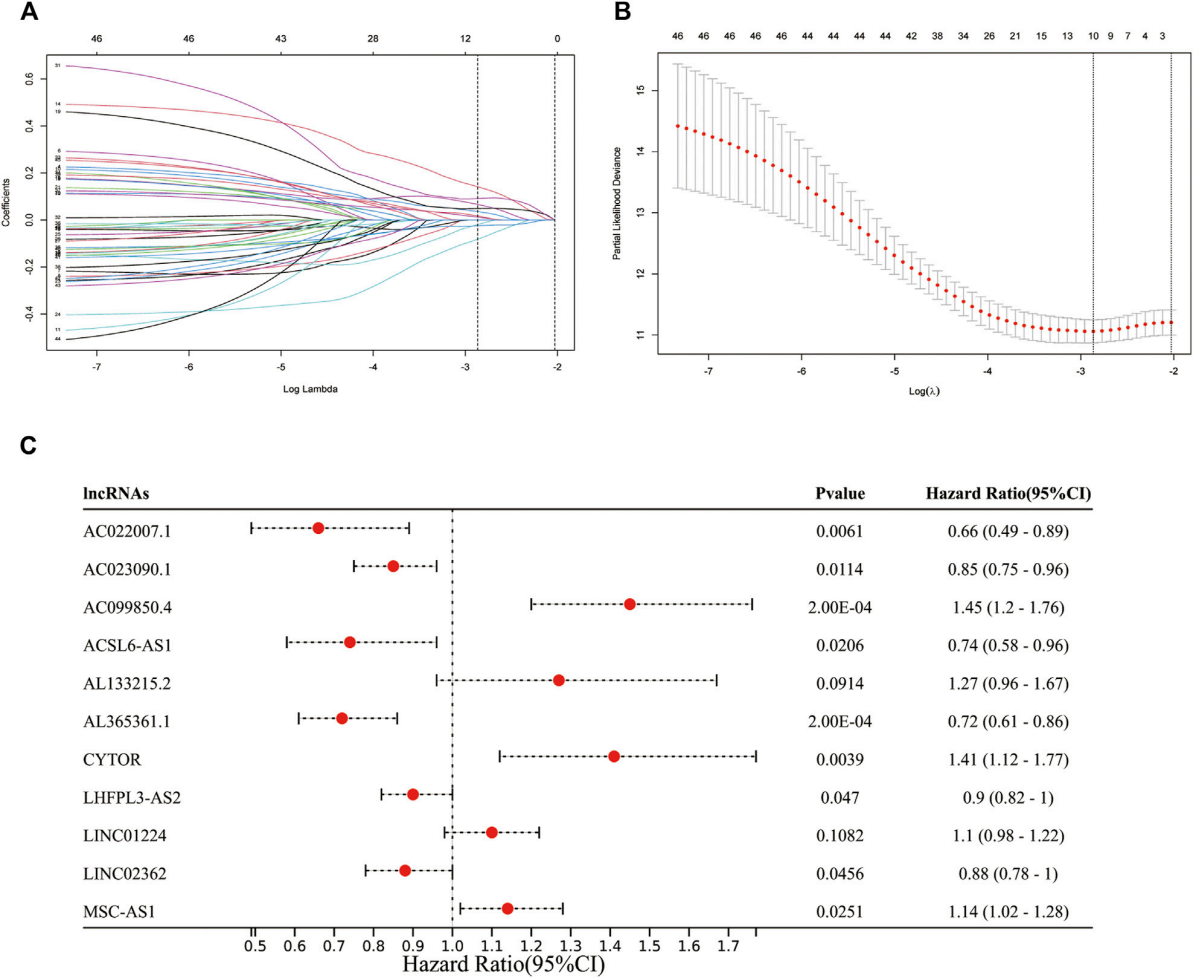
Identification of prognostic mitochondrial-related and ferroptosis-related lncRNA. **(A,B)**: 11 MF-related prognostic lncRNAs based on the min lambda. **(C)**: Forest plot showed the results of the multivariate Cox regression.

Also, these MF-related lncRNAs were identified as potential prognostic indicators and were subjected to least absolute shrinkage and selection operator (LASSO) logistic regression algorithm analysis in the training cohort. Moreover, 10-fold cross-validation was also used for cross-validation. We identified 15 MF-related prognostic lncRNAs based on the min lambda (Figures 3A,B). Some 15 MF-related prognostic lncRNAs were enrolled into multivariate Cox regression analysis to further identify the prognostic lncRNAs. Finally, AC022007.1, AC023090.1, AC099850.4, ACSL6-AS1, CYTOR, LHFPL3-AS2, AL365361.1, LINC02362, and MSC-AS1 were regarded as the MF-related and prognostic lncRNA (Figure 3C).

### Validated the Expression of Mitochondrion and Ferroptosis-Related Prognostic Long Non-Coding RNA in the Independent Cohort

**FIGURE 4:**
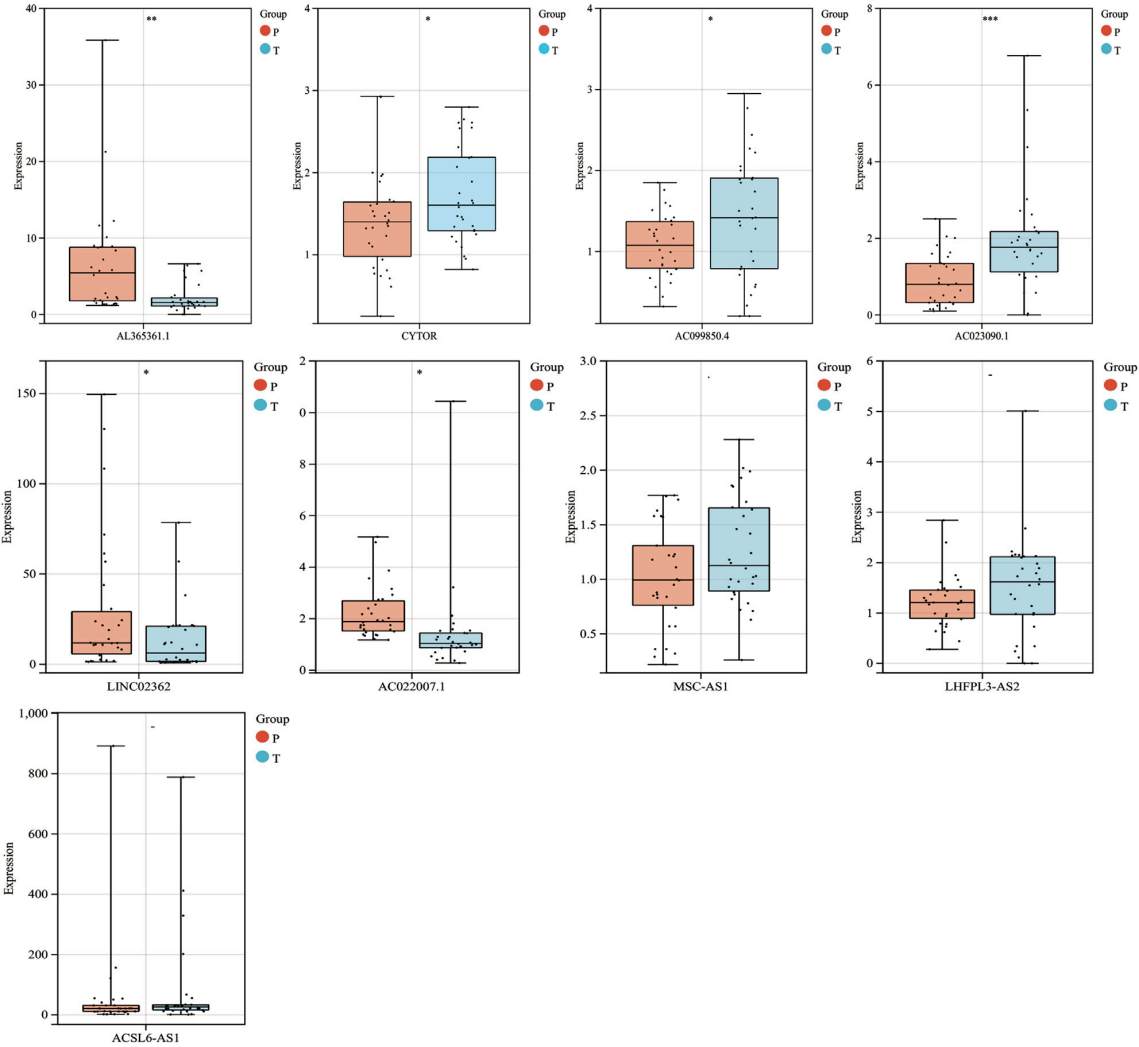
mRNA expression levels of 9 MF-related prognostic lncRNAs in HCC from the GPDH cohort. *p < 0.05.**p < 0.01.***p < 0.001 “-” means nonsignificant.

To further validate these prognostic lncRNA expression experimentally, 30 pairs of hepatocellular carcinoma tissues and adjacent normal liver tissues were selected from Guangdong Provincial People’s Hospital and detected the nine lncRNA expression by RT-qPCR. We found that AC023090.1, AC099850.4, and CYTOR were significantly highly expressed in HCC tissues, while the expression of AL365361.1, AC022007.1, and LINC02362 was largely decreased in HCC tissues. The expression levels of MSC-ASL, LHFPL3-AS2, and ACSL6-AS1 were upregulated in the HCC sample though without statistically significant (Figure 4).

### Construction and Evaluation of a Mitochondrion and Ferroptosis-Related Long Non-Coding RNA Prognostic Signature for LIHC Patients

**FIGURE 5:**
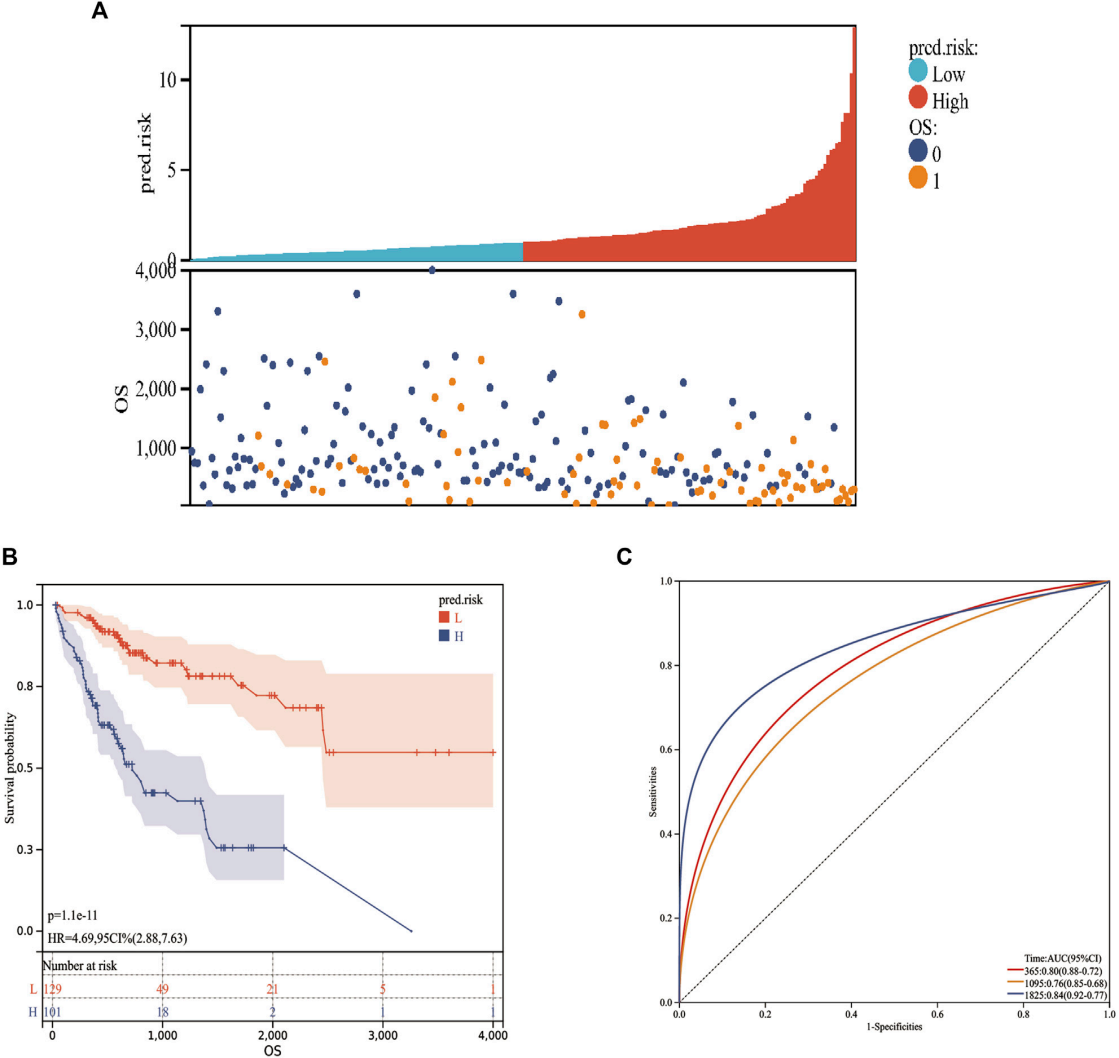
Prognostic values of the MF-related lncRNA signature in training cohort. **(A)**: Risk score. **(B)**: Kaplan–Meier curve between high‐ and low‐risk groups. **(C)**: time‐ROC curve of prognostic MF-related lncRNA signature in the training cohort. 0: Alive; 1: Dead; L: Low-risk; H: High-risk.

Considering the expression of AC022007.1 in the TCGA cohort was opposite to the validation results of the GDPH cohort, a MF-related LPS was built based on the AC023090.1, AC099850.4, ACSL6-AS1, CYTOR, LHFPL3-AS2, AL365361.1, LINC02362, and MSC-AS1. The risk scores were calculated in the training cohort. The risk score of each patient could be calculated by the following formula: risk score = (−0.16416 × expression value of AC023090.1) + (0.37193 × expression value of AC099850.4) + (−0.29628 × expression value of ACSL6-AS1) + (−0.32870 × expression value of AL365361.1) + (0.34095 × expression value of CYTOR) + (−0.10062 × expression value of LHFPL3-AS2) + (−0.12666 × expression value of LINC02362) + (0.13118 × expression value of MSC-AS1). According to the best cutoff value of the risk scores, the training cohort can be divided into low-risk and high-risk groups (Figure 5A). Kaplan–Meier analysis was utilized to access the stratification power of the MF-related LPS. The OS curve indicated that compared with low-risk group, the high-risk group had poorer OS (Figure 5B). Moreover, by using the time-dependent ROC curves, the MF-related LPS showed excellent accuracy in predicting 1-,3-, and 5-year OS (1-year AUC = 0.80 3-year AUC = 0.76, 5-year OS = 0.84, Figure 5C).

### Mitochondrion and Ferroptosis-Related Long Non-Coding RNA Prognostic Signature was an Independent Risk Factor for Overall Survival of the Training Cohort

**FIGURE 6:**
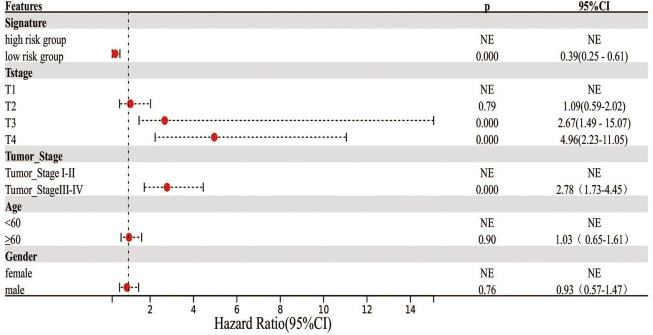
Cox regression analysis on the clinicopathological factors and MF-related lncRNA signature. Forest plot showed the results of univariate Cox regression.

Cox regression analysis was performed to identify the prognostic factors for LIHC patients. After removing the patients with unknown information and performing the univariate Cox regression analysis, MF-related LPS, tumor stage, and T stage were regarded as potential risk factors (Figure 6). All significant univariable predictors were enrolled into multivariate Cox regression analysis. Finally, the results indicated that MF-related LPS and tumor stage were independent prognostic factors for overall survival (OS) in patients with hepatocellular carcinoma who underwent surgical therapy (Table2).

**TABLE 2:** Multivariate Cox regression in the training cohort.

Features	HR	HR.95%L	HR.95H	*p* value	Hazard Ratio (95% CI)
Signature					
High-risk group				NE	
Low-risk group	0.39	0.25	0.61	<0.01	0.39 (0.25–0.61)
Tumor_stage					
I–II				NE	
III–IV	2.47	1.69	3.62	<0.01	2.47 (1.69–3.62)

### Validation of Mitochondrion and Ferroptosis-Related Long Non-Coding RNA Prognostic Signature in the Validation Cohort

**FIGURE 7:**
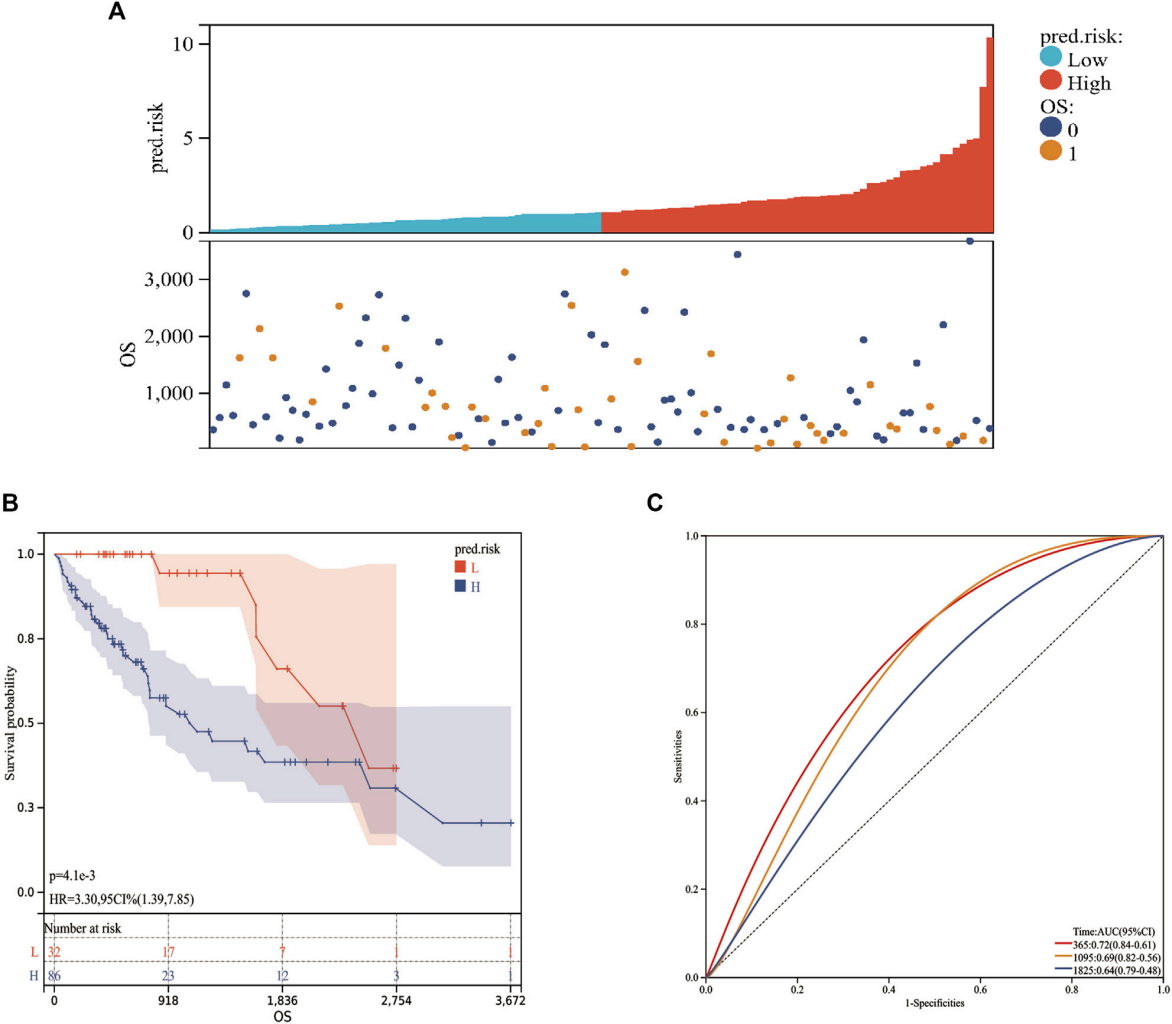
The prognostic values of MF-related lncRNA signature in validation cohort. **(A)**: Risk score. **(B)**: Kaplan‐Meier curve between high- and low-risk groups. **(C)**: Time‐ROC curve of prognostic MF-related lncRNA signature in the validation cohort.0: Alive; 1: Dead; L: Low-risk; H: High-risk.

Based on the MF-related LPS and the expression of each lncRNA from the validation cohort, the predicted risk scores were calculated. According to the best cutoff value from the training cohort, the validation population can also be divided into low-risk and high-risk groups (Figure 7A). Kaplan–Meier analysis validated the perfect stratification power of the MF-related LPS. The OS curve indicated that compared with the low-risk group, patients in the high-risk subgroup had worse survival (Figure 7B). As illustrated in Figure 7C, the AUC of MF-related LPS showed excellent accuracy in predicting 1-,3-, and 5-year OS (1-year AUC = 0.72, 2-year AUC = 0.69, 5-year OS = 0.64). After performing the multivariate Cox regression analysis, the MF-related LPS was validated as the independent prognostic factors for overall survival (OS) in the validation cohort (Table 3).

**TABLE 3:** Multivariate Cox regression analysis in the validation cohort.

Features	HR	HR.95%L	HR.95%H	*p* value	Hazard Ratio (95% CI)
Signature					
High-risk group				NE	
Low-risk group	0.33	0.13	0.82	0.016	0.33 (0.13–0.82)
Tumor stage					
I–II				NE	
III–IV	1.06	0.14	7.96	0.96	1.06 (0.14–7.96)
Tstage					
T1				NE	
T2	1.62	0.85	3.07	0.14	1.62 (0.85–3.07)
T3	3.04	0.37	25.34	0.31	3.04 (0.37–25.34)
T4	1.06	0.04	11.81	0.79	1.06 (0.04–11.81)

### The Association Between Mitochondrion and Ferroptosis-Related Long Non-Coding RNA Prognostic Signature and Clinicopathological Characteristics

We next performed association analysis to determine whether the MF-related LPS was related to clinicopathological characteristics in whole TCGA HCC patients. As Table 4 indicated, there is no difference in age and gender between the high-risk group and low-risk group. But, there are more patients in the low-risk group with AJCC stage I–II, while there are more patients in the high-risk group with AJCC stage III–IV. As for T stage, 130 patients at T1 stage are in the low-risk group and 77 patients at T2–T3 stage are in the high-risk group. Also, to further investigate the prognostic values of MF-related LPS in different subtypes of the TCGA LIHC population, subgroup analysis was conducted. The results revealed that the high-risk group is related to poorer OS in patients at early AJCC stage (I–II) or advanced AJCC stage (III–IV), patients with tumor stage T1, T2, or T3, and patients with age ≥60 or <60 (Supplementary Figure S1).

**TABLE 4:** Clinical characteristics of HCC patients in the high-risk group and low-risk group.

Characteristics	Low (*n* = 225)	High (*n* = 123)	*p* value
T stage			0.000041
T1	130 (57.78%)	39 (31.71%)	
T2	43 (19.11%)	43 (34.96%)	
T3	43 (19.11%)	34 (27.64%)	
T4	6 (2.67%)	7 (5.69%)	
unknown	3 (1.33%)	0 (0.0e + 0%)	
N stage			0.24
N0	152 (67.56%)	91 (73.98%)	
N1	3 (2.33%)	0 (0.0e + 0%)	
NX	70 (30.11%)	32 (26.02%)	
M stage			0.13
M0	154 (68.44%)	94 (76.42%)	
M1	4 (1.78%)	0 (0.0e + 0%)	
MX	67 (29.78%)	29 (23.58%)	
Tumor_stage			3.10E-04
stage I	123 (54.67%)	39 (31.71%)	
stage II	40 (17.78%)	38 (30.89%)	
stage III	45 (20%)	37 (30.08%)	
stage IV	4 (1.78%)	0 (0.0e + 0%)	
unknown	13 (5.77%)	9 (7.32%)	

### Differentially Expressed Genes Between High- and Low-Risk Groups Were Identified and Function Enrichment Analysis Were Performed

**FIGURE 8:**
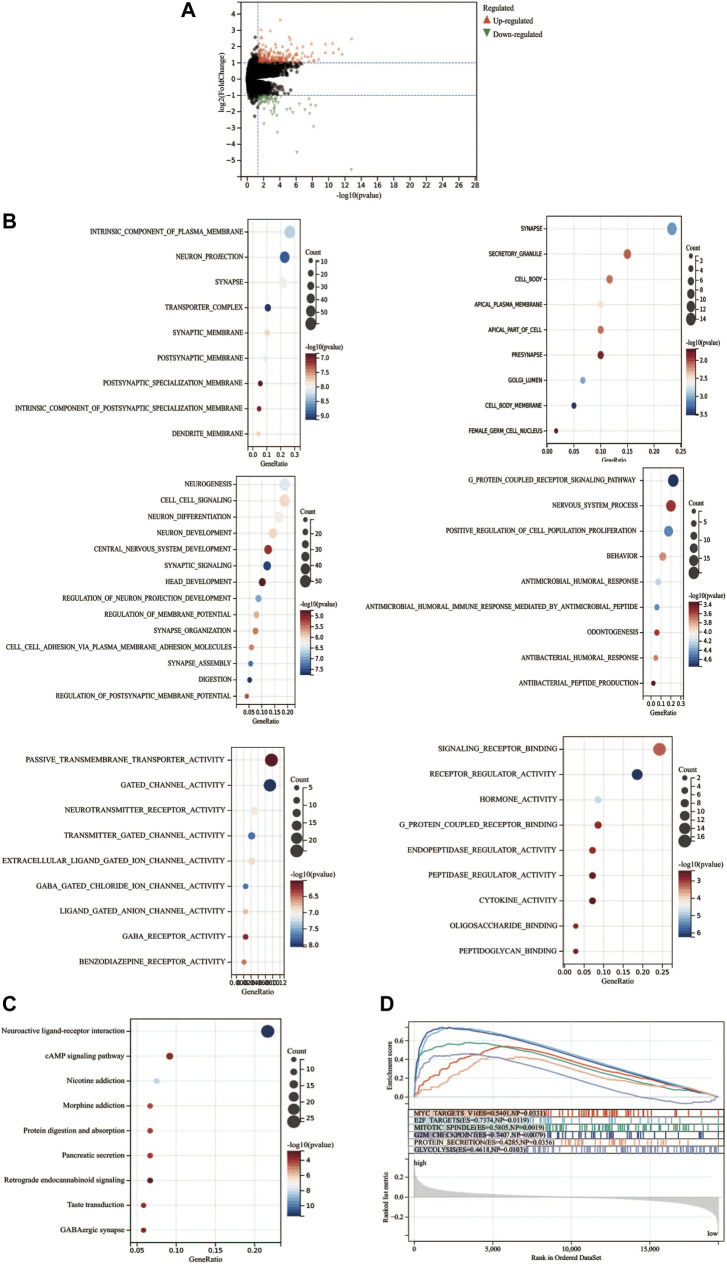
Functional enrichment analysis of differentially expressed genes. **(A)** Differentially expressed genes between high- and low-risk groups. **(B)** GO analysis of the differential expression genes. **(C)** KEGG analysis of the differential expression genes. **(D)** Gene set enrichment analysis.

To further investigate the differences between high- and low-risk groups, differential expression analysis was conducted by the *Deseq2* package. A total of 408 DEGs (upregulation: 308 and downregulation: 100) between two groups were identified (*p* < 0.05, Log2FoldChange>1.0, Figure 8A). Moreover, the DEGs between the high-risk and low-risk groups were used to perform GO enrichment and KEGG pathway analyses. GO component enrichment analysis showed that upregulated DEGs may be involved in “intrinsic component of plasma membrane, neuron projection, synapse, transporter complex,” etc., while downregulated DEGs may be involved in “synapse, secretory granule cell body,” etc. (Figure 8B). The GO biological process indicated that upregulated DEGs were mainly enriched in “neurogenesis, cell signaling, regulation of membrane potential,” etc., while the downregulated DEGs were mainly enriched in the “G protein-coupled receptor signaling pathway, positive regulation of cell population proliferation, antimicrobial humoral response,” etc. The GO molecular function indicated that upregulated DEGs may be involved in “passive transmembrane transporter activity, gated channel activity, neurotransmitter receptor activity,” etc. The downregulated DEGs were mainly enriched in “signaling receptor binding, receptor regulator activity, and hormone activity.” KEGG pathway analyses indicated that these upregulated genes were mainly involved in “neuroactive ligand-receptor interaction, cAMP signaling pathway, nicotine addiction, and retrograde endocannabinoid signaling.” (Figure 8C). GSEA analysis indicated that the “G2M checkpoint, E2F targets, MYC targets, GLYCOSIS signaling, etc.” were activated (Figure 8D).

### Correlation Between the Mitochondrion- and Ferroptosis-Related Long Non-Coding RNA Prognostic Signature and Immune Cell Infiltration

**FIGURE 9:**
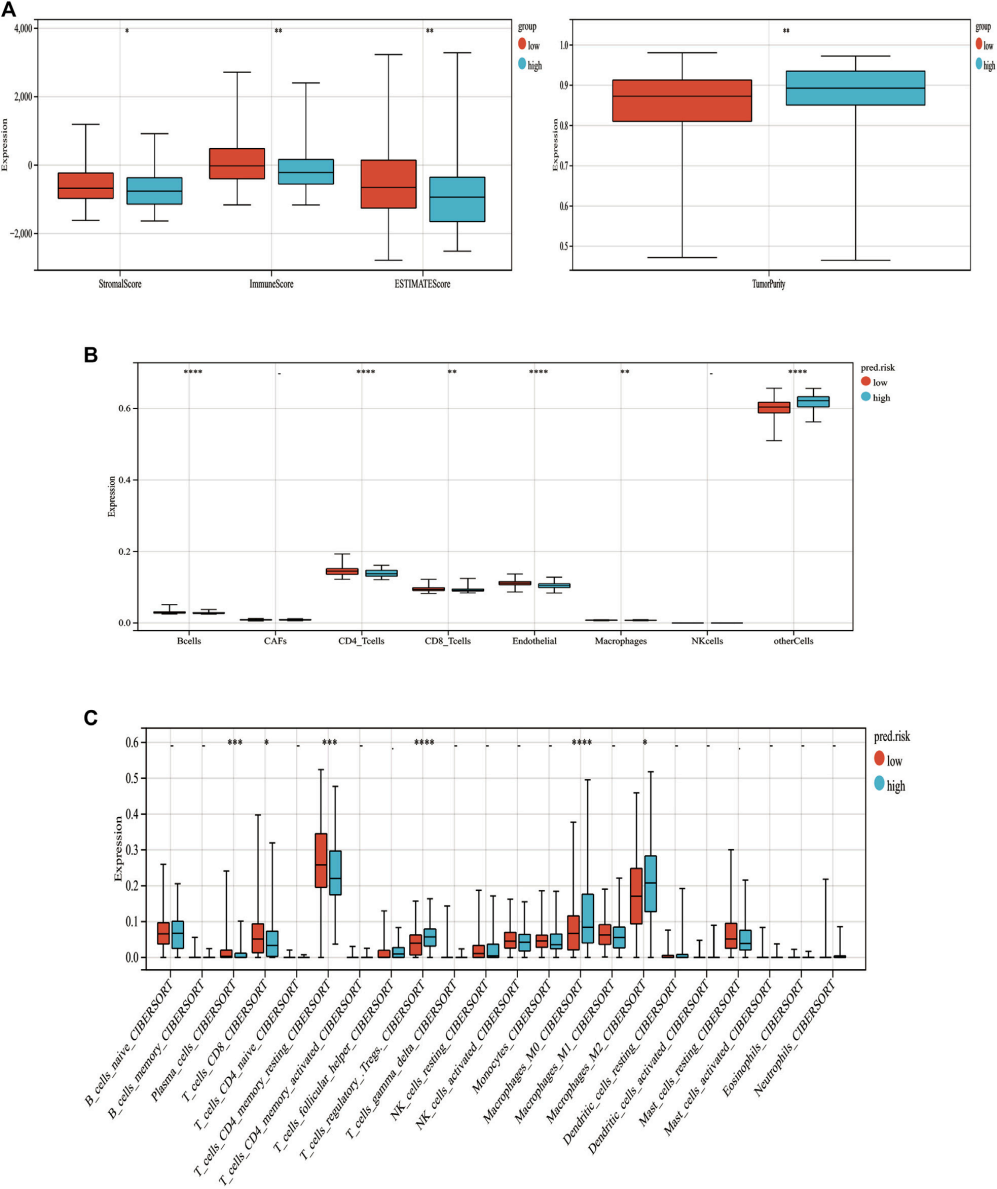
Correlation between tumor-infiltrating immune cells and MF-related lncRNA signature. **(A)** Immune, stromal, STIMATE scores and tumor purity within the low‐ and high‐risk groups. **(B)** Relations between immune cell types and different risk groups based on EPIC analysis. **(C)** Relations between immune cell types and different risk groups based on CIBERSORT analysis. *p < 0.05. **p < 0.01. ***p < 0.001. ***p < 0.0001.

To assess the relationship between immune cell infiltration and our MF-related LPS, we performed the ESTIMATE analysis. As shown in Figure 9A, the high-risk group had lower ESTIMATE score and immune score but had higher tumor purity than the low-risk group. Therefore, the EPIC analysis was performed to investigate the immune cell infiltration. As shown in Figure 9B, there were several different immune cell infiltrations between the high-risk and low-risk groups. The low-risk group had much more B cells, macrophages, CD 8+ T cell, and CD 4+ T cell infiltration than the high-risk group. To further explore the correlation between the MF-related LPS and the infiltration of immune cell subtypes, the CIBERSORT algorithm was used. The results showed that the low-risk group had significant correlation with plasma cells, T cells, CD8 T cells, CD4 memory resting, while the high-risk group had significant correlation with regulatory T cells and M0 and M2 macrophages (Figure 9C).

## Discussions

Hepatocellular carcinoma is a common malignancy of the digestive system with difficulty in early diagnosis, high recurrence rate, and poor long-term survival. Therefore, it is urgent to figure out new therapeutic targets for hepatocellular carcinoma. LncRNAs are the important types of noncoding RNAs and can promote tumorigenesis, cell proliferation, migration, and metastasis by regulating gene expression on epigenetic modification. A growing number of studies show that mitochondrial dysfunction and ferroptosis are closely related to tumor progression. However, there is lack of systematic studies on the relations among lncRNAs, ferroptosis, and mitochondrial dysfunction and the mechanism of how to impact the fate of tumor cell and the immune cells.

In our study, we first obtain the mitochondria-related gene and ferroptosis-related genes from the previous studies. Also, after performing the Pearson analysis, the lncRNAs with the most significantly prognostic value and strongest correlation with mitochondria and ferroptosis were identified. The MF-related lncRNA-associated mitochondrial function defects and ferroptosis were further screened by Lasso-Cox regression.

Eight lncRNAs, namely, AC023090.1, AC099850.4, AL365361.1, CYTOR, ACSL6-AS1, LHFPL3-AS2, LINC02362, and MSC-AS1 were identified as the independent prognostic lncRNAs. Moreover, we verified the expression of these eight lncRNAs through RT-qPCR in our 30 pair samples from the GDPH cohort.

Previous study pointed out that AC099850.4 was one of the lncRNAs with most connections to miRNA or mRNA in High-Grade Serous Ovarian Cancer (Zhao et al., 2020). AL365361.1 was reported to improve the survival of head and neck squamous cell carcinoma, contributing to modification of the tumor microenvironment (Zhong et al., 2020). CYTOR and MSC-AS1were highly expressed in many malignancies (Chen et al., 2021a; Chen et al., 2021b; Liu et al., 2021). Further studies had shown that CYTOR and MSC-AS1inhibited the apoptosis of tumor cells and promoted EMT and tumor proliferation by acting as the ceRNAs for the miRNAs, such as miR-4775, miR-125B-5P (Hu et al., 2020; Chen et al., 2021b; Wang and Zhang, 2021; Liang et al., 2022). However, AC023090.1, ACSL6-AS1, LHFPL3-AS2, and LINC02362 are not mentioned in the previous research studies.

Based on these eight lncRNAs, we established the MF-related LPS. This LPS had very outstanding stratification ability. Patients can be divided into the high-risk group and low-risk group according to the risk scores calculated by the LPS. After further analysis, patients in the high-risk group tended to be at advanced T stage and AJCC stage and even with poorer overall survival. We also conducted the subgroup analysis, and LPS also had excellent stratification capabilities in different age, gender, T stage, and AJCC stage. We also found that LPS had very outstanding prognostic predictive capabilities, and 5-year AUC was 0.84. Moreover, based on 118 patients from TCGA cohorts, the stratification ability and prognostic prediction capability were fully validated.

Further functional enrichment analysis on the DEGs suggested that overexpressed genes in high-risk groups were associated with the signaling between cell and cell. GSEA results showed that the pathways related to tumorigenesis and tumor progression were significantly enriched in the high-risk group. These results indicated that these lncRNAs may affect mitochondria functions and ferroptosis in HCC through the cell cycle pathways.

Increasing immunosuppressive cells is one of the immune escape mechanisms which would make CD8^+^ T cells unable to identify the cancer cells and lead to tumor progression. Therefore, we analyzed the different immune cell infiltration between these two groups. Our studies have shown that these two groups had different immunocyte infiltration and tumor microenvironments. In our study, the infiltration ratio of regulatory T cells and M0 and M2 macrophages was higher in the high-risk group and the infiltration ratio of T cells CD8, CD4 memory resting, and monocytes was higher in the low-risk group. Studies reported that CD4 + T-cell help could enhance the response of CTLs (Borst et al., 2018) and patients with high CD4^+^ T cells infiltration were associated with better survival (Reyes et al., 2013). Mark Farha pointed out that the M0 macrophage-enriched group was a poor prognostic factor in HCC patients (Farha et al., 2020). Previous research studies revealed that M2 macrophages could promote the tumorigenesis by affecting the tumor microenvironment (Neophytou et al., 2021). A recent study indicated that a large number of Treg cells in the tumor microenvironment could inhibit the antitumor functions of human CD8^+^ T cells (Maeda et al., 2014). Also, high infiltration of Treg cells, in tumor microenvironment was related to poor prognosis (Tanaka and Sakaguchi, 2017).

In summary, our results suggested that AC023090.1, AC099850.4, AL365361.1, CYTOR, ACSL6-AS1, LHFPL3-AS2, LINC02362, and MSC-AS1 had the potential to be significant biomarkers. Also, we speculated these lncRNAs were regulating the mitochondria functions and ferroptosis through specific signaling pathways we mentioned above, which in turn influenced the tumorigenesis, cell proliferation, and differentiation. On the other hand, our research indicated that these lncRNAs are directly or indirectly regulating the tumor microenvironment by altering the infiltration or differentiation of immune cells and promoted tumor progression.

Despite the important findings, there is no denying that this study has several limitations. First, the data were collected from the online public datasets with few clinicopathological data, potential errors, or deviations that should be considered. Second, some verification results in the GDPH cohort would not be statistically significant because of the small sample size, and further independent and large number cohorts are needed to validate our findings in future. In addition, the molecular mechanisms on how these lncRNAs function must be explored further through fundamental experiments.

## Conclusion

To conclude, based on the nine mitochondria and ferroptosis-related lncRNAs, a MF-related LPS was built successfully. More importantly, we found that these lncRNAs may affect mitochondrial functions, ferroptosis, and immune cell infiltration in HCC through specific pathways, which may provide valuable insight on the progression and therapies of HCC.

## Data Availability

The datasets TCGA (https://portal.gdc.cancer.gov/) for this study can be found in the websites. And further inquiries can be directed to the corresponding authors.

## Supplementary Material

Supplementary Figure S1 Association between MF-related LPS and clinicopathological characteristics.
