# Supplementary figures and images for: Development and Validation of a Novel Mitochondrion and Ferroptosis-Related Long Non-Coding RNA Prognostic Signature in Hepatocellular Carcinoma
Source: Front Cell Dev Biol. 2022 Aug 12;10:844759. doi: 10.3389/fcell.2022.844759 (PMC9413087; doi:10.3389/fcell.2022.844759)

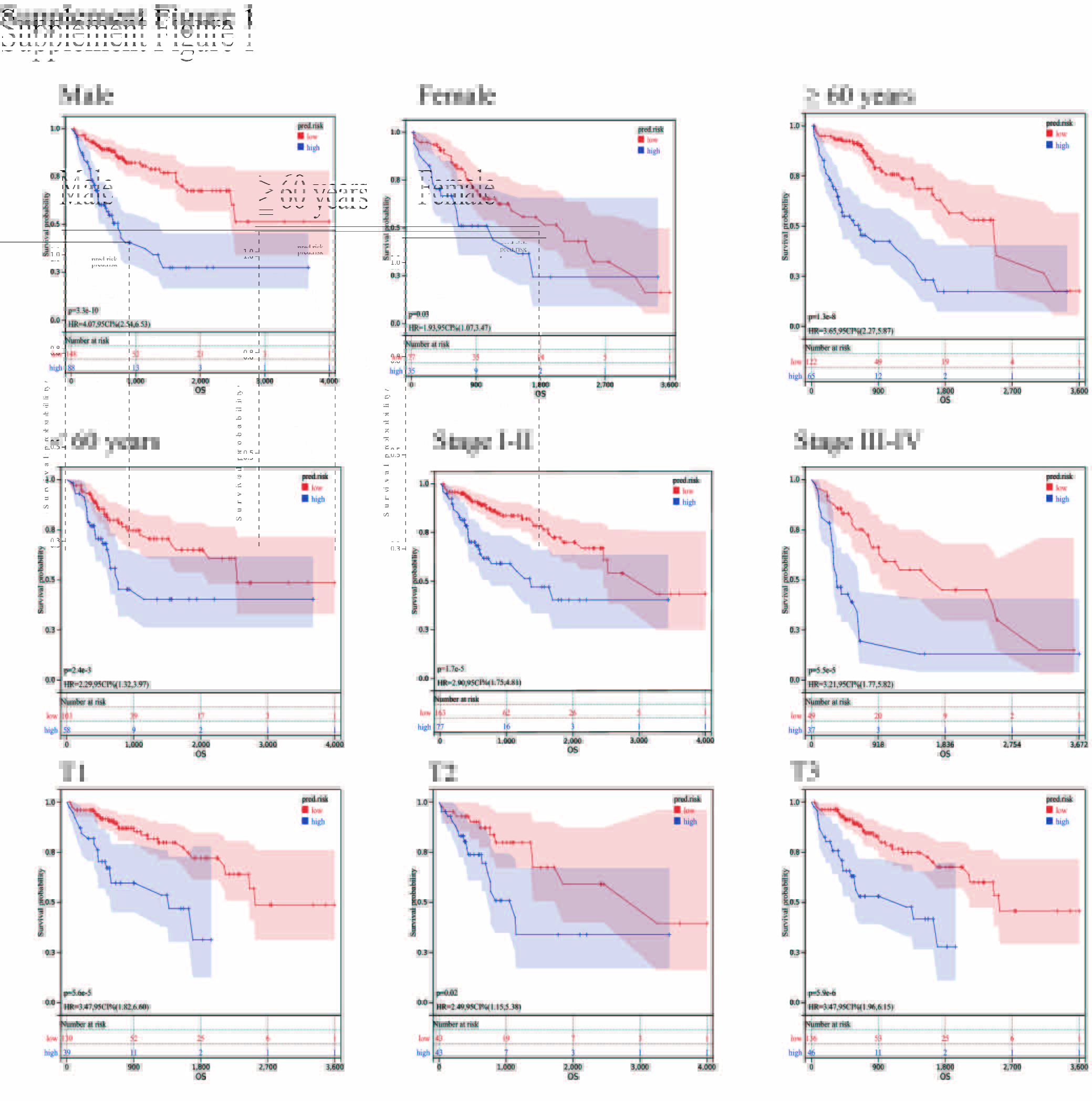

Supplement: Supplementary file 3 [file Image1.JPEG]
